# Correlation of the position and status of the polar body from the fertilized oocyte to the euploid status of blastocysts

**DOI:** 10.3389/fgene.2022.1006870

**Published:** 2022-09-20

**Authors:** Yongle Yang, Wei Tan, Changsheng Chen, Lei Jin, Bo Huang

**Affiliations:** ^1^ Reproductive Medicine Center, Tongji Hospital, Tongji Medical College, Huazhong University of Science and Technology, Wuhan, China; ^2^ Wuhan Huchuang Union Technology Co.,Ltd, Wuhan, Hebei, China

**Keywords:** polar body, euploidy, aneuploidy, mosaicism, blastocyst

## Abstract

Polar bodies are tiny cells that are extruded during oocyte meiosis and are generally considered not essential for embryonic development. Therefore, polar bodies have been widely used as important materials for the preimplantation genetic diagnosis of human embryos. Recent studies have shown that polar bodies mediate embryonic development and that their morphology is related to embryo quality and developmental potential. However, the relationship between the emission of the polar body and embryonic euploidy remains unclear. In this study, a total of 1,360 blastocyst trophectoderm (TE) biopsies were performed, and blastocyst ploidy results were correlated with the state of polar bodies. The results showed that polar body angle size and polar body status are not directly related to whether the blastocysts are euploid, aneuploid, or mosaic (*p* > 0.05). Therefore, in the process of clinical embryo selection, embryologists should not predict the euploidy of blastocysts based on the state of polar bodies, thus affecting embryo selection.

## Introduction

Asymmetric cell division, in which a precursor cell produces two progeny of different sizes and/or different developmental fates, is as common as cell division itself the course (Liu et al., 2012), throughout development in all animals (Knoblich et al., 2001), an extreme example of asymmetric cell division is meiosis in the animal ovary. To produce robust haploid oocytes, meiosis I expel half of the homologous chromosomes, and meiosis II expels half of the sister chromatids, in the form of small, inanimate cells called the first and second poles, respectively. Thus, female meiosis produces a haploid mature oocyte next to two polar bodies (or sometimes three if the first polar body divides further).

During early embryogenesis, primordial germ cells migrate into the developing gonads. Once there, these cells (called lecithin in female fetuses) proliferate through mitotic cell division and then begin meiosis I, but are stopped (a G_2_-like arrest) at the end of prophase as primary oocytes. Two biochemical events during the fetal stage of meiosis have important implications for the generation of oocyte aneuploidy in adult females (Baltus et al., 2006). The first is sister chromatid cohesion, established during the meiotic S phase by a circular protein complex called cohesin around the entire length of the sister chromatid (Uhlmann et al., 1998; Revenkova et al., 2010). The second is homologous recombination (crossover) between non-sister chromatids shortly after (prophase synaptic stage). A combination of sister chromatid cohesion and non-sister crossover links homologous chromosomes together; this link persists for decades in women. During oocyte maturation, which occurs with each ovulatory cycle, cohesin is degraded/removed from the chromosome arms, resulting in the segregation of homologous chromosomes (Kudo et al., 2006). Sister chromatids separate during fertilization (meiosis II) when cohesin is degraded (Lee et al., 2006). Therefore, the polar body Ⅱ is homologous to the chromosome of the oocyte, which with the same genetic and reproductive functions as the oocyte cell. In clinical practice, polar bodies have been used in the fields of maternal infertility and early diagnosis of abnormal chromosomal genetic diseases (Verlinsky et al., 1997; Briggs et al., 2000; Durban et al., 2001; Gutiérrez-Mateo et al., 2004).

The polar body is one of the objective criteria for evaluating the quality of oocytes. There is a close relationship between the morphology of the first polar body and oocyte quality. Cut correlation, smooth, fragment-free, non-degenerated polar bodies tend to often predict high-quality oocytes and good developmental potential (Lasiene et al., 2009). There is a correlation between the irregularity of the shape of the first polar body of the oocyte cell and the number of embryos blastomeres, the proportion of fragments, and the clinical pregnancy rate. It is proposed that the combination of the first polar body shape evaluation is conducive to the selection of embryos with good quality for transfer, to improve clinical pregnancy rates (Navarro et al., 2009). Other studies have shown that irregular polar body morphology is associated with poor plasma embryos are significantly correlated (Ubaldi et al., 2008). New research shows that polar bodies may play a certain regulatory role in mouse embryonic development (The second polar body contributes to the fate asymmetry in the mouse embryo) (Jin et al., 2022). Numerous studies have shown that the state of the polar body is related to the quality and developmental potential of the embryo. The polar body may regulate the development of the embryo, rather than the traditional understanding that the polar body is a small cell with no function {Verlinsky et al., 2003; Lasiene et al., 2009; Navarro et al., 2009; Ubaldi et al., 2008}.

Therefore, it is one of the key issues that the majority of reproductive medicine workers pay attention to do more research on polar body and clarify the role of polar body. Therefore, this study starts from the relationship between the state of polar bodies and the euploidy of embryos, in toe the Time-lapse (TL) system to judge the euploidy of embryos and predict the developmental potential of embryos by observing the state of polar bodies.

## Materials and methods

### Patients and experimental design

Due to the observational nature of retrospective studies, it should not be assumed that there is a causal relationship between the euploidy of the embryo with the status of the polar body; controlled experiments need to be carefully designed before providing clinical recommendations. In this study, we retrospectively evaluated the status of polar body and euploidy of embryo that could be considered for transfer by next-generation sequencing (NGS) analysis among patients undergoing preimplantation genetic testing (PGT) treatment (Chen et al., 2021) between January 2020 and December 2021 at the Reproductive Medicine Center of Tongji Hospital. Only embryo biopsies in fresh cycles were considered to eliminate the effects of vitrification. This research was conducted at the Reproductive Medicine Center of Tongji Hospital, Tongji Medical College, and Huazhong University of Science and Technology. All procedures and protocols were approved by the ethics committee. The study included 1,360 embryos from 448 patients. We select blastocysts that can be used for the transfer to trophectoderm (TE) biopsy, observe the state of polar bodies of these embryos, and then count the potential relationship between different states of polar body and embryo euploidy.

### Embryo culture

Embryos were cultured according to conventional methods. After the oocytes were collected, the cumulus-oocyte complex was cultured in a fertilization medium (G-IVF; Vitrolife). A discontinuous gradient solution (45 and 90%, SpermGrad, Vitrolife) was used to wash the spermatozoa, and the obtained sperm pellet was placed at the bottom of the fertilization medium. The cumulus-oocyte complex was in contact with human recombinant hyaluronidase (80 IU/ml) for a short time, and then the granulosa cells were peeled off by mechanical action. After intracytoplasmic sperm injection (ICSI), the oocytes were transferred to the prepared TL imaging dish and cultured in the TL system. Embryo culture was performed in the integrated embryo culture TL system (Embryo Scope plus; Vitrolife), in which images are taken every 10 min on seven different focal planes. All embryos were cultured in a culture device under the same conditions.

### Polar body observation

**FIGURE 1:**
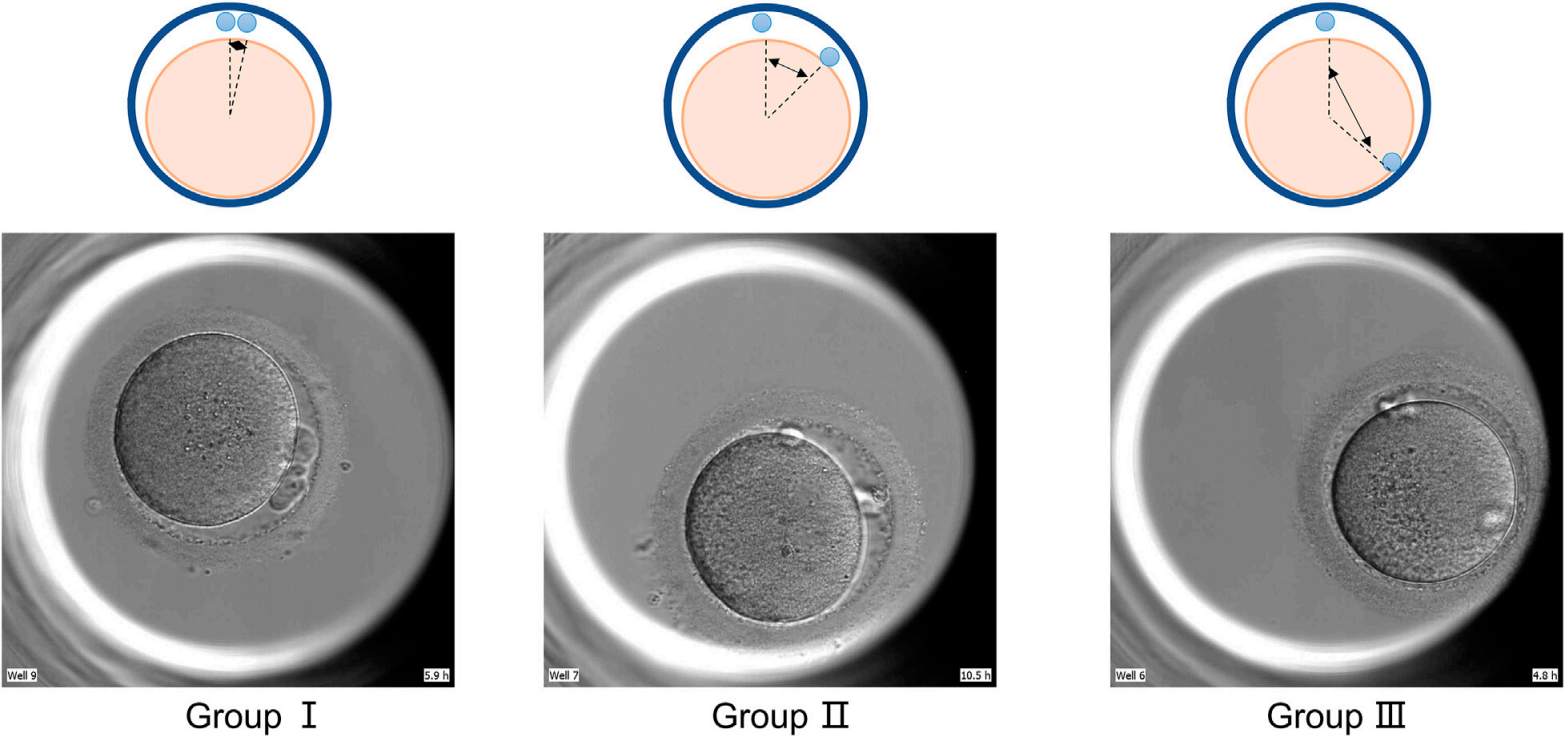
Group by polar body position. GroupⅠ: pole body angle≤30°; GroupⅡ: polar body angle between 30° and 90°; GroupⅢ pole body angle ≥90°.

We divide the state of the polar body into two cases. The first one is based on the positional relationship between the two polar bodies, which is divided into three groups (as show in Figure 1: group Ⅰ ≤30°; group Ⅱ 30°–90°; group Ⅲ ≥90°) according to the size of the included angle. The second is divided into five groups according to the shape of the two polar bodies: in group A: both polar bodies are complete spherical; in group B: one polar body is completely spherical and the other is fragmented; in group C: both polar bodies are fragmented; group D: one of the two polar bodies is irregular in shape; group E: both polar bodies are irregular in shape. The time when the second polar body is excluded as the time when the polar body state is observed.

### Clinical TE biopsy

Details of the embryo culture procedures used have been previously described (Huang et al., 2014; Huang et al., 2015). Briefly, all embryos were fertilized by ICSI, and those showing normal fertilization, more than four blastomere cells, and ≤20% fragments on Day 3 were cultured to the blastocyst stage. On Day 5 or 6, a TE biopsy was performed on the blastocysts with a grade ≥3BC (Gardner et al., 1999) to obtain a sample of 5–10 TE cells. The biopsy procedure was the same as that used by Yang (Yang et al., 2020). After the biopsy, the obtained samples were loaded into 200 μL sampling tubes (YiKon, Suzhou, China) and stored at -20 °C waiting for genetic testing, and the blastocysts were vitrified immediately using a Kitazato vitrification kit (Kitazato Biopharma Co. Ltd, Shizuoka, Japan).

### NGS analysis

We used multiple annealing and looping-based amplification cycles (MALBACs) based single-cell whole-genome amplification (WGA) protocol to amplify the samples following the commercial kit protocol from Yikon Genomics (Zong et al., 2012). After a series of DNA fragmentation, amplification, tagging, and purification, the products were purified using a size selection and pooled with an equal quantity of each sample. The final library was sequenced using Life Technologies Ion Proton platform at approximately 0.04× genome depth. This sequencing throughput yields reproducible copy number variation (CNV) with approximately 4 MB resolution to detect the variation. The threshold for aneuploidy detection was set at > 70% while the threshold for mosaicism detection varied by chromosomes. For Chromosomes 13, 16, 18, and 21, the lower limit was 30%, and for Chromosome 19 the lower limit was 50%, and for the others, it was 40% (Wu et al., 2021; Chuang et al., 2020). Values under the lower limit were indicative of euploidy.

### Statistical analysis

Categorical variables were expressed as percentages, chi-square tests were utilized, and Fisher’s exact tests were used when necessary. Logistic regression analysis was used to explore the influence of biopsy doctors, embryo euploidy and polar body angle, and polar body status. Origin 9.0 software was used (Origin Lab, Northampton, United States ), and *p* < 0.05 was considered statistically significant.

## Results

### General outcomes

This study included patients who underwent PGT cycles at our center from January 2020 to December 2021. We studied embryos used for a biopsy on day 5 or six in all patients. Biopsy results classified all embryo euploidy into three categories: euploid, aneuploid, and mosaic, accounting for 50, 40.8 and 9.2%, respectively. All embryo euploidy used for biopsy were matched to their polar body status and were used to assess the relationship of polar body status to euploidy.

### The relationship between the relative positions of polar bodies and their embryonic euploidy

**FIGURE 2:**
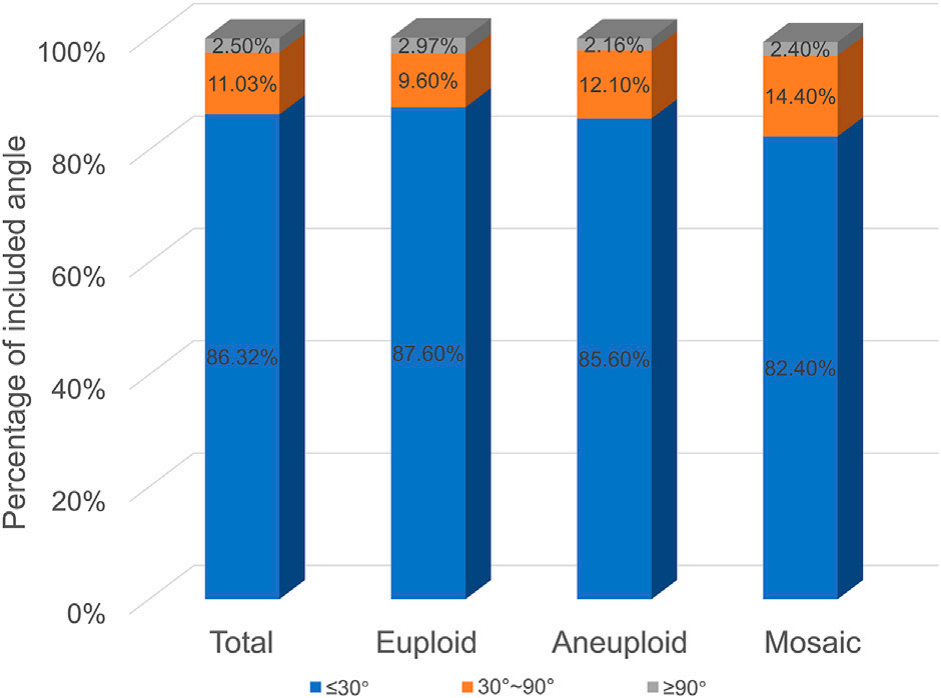
Polar body angle and embryo euploidy.

As shown in Figure 2, the distribution of the three euploidy was the same in the three groups with different included angle ranges. The smaller included angle accounted for the largest proportion, exceeding 80% in all three groups, and the proportion of embryos decreased as the included angle increased (In the euploid group, the angles of polar bodies from small to large accounted for 87.6, 9.6, and 2.97%, respectively. In the aneuploid group, the angles of polar bodies from small to large accounted for 85.6, 12.1, and 2.16%, respectively. In the mosaic group, the angles of polar bodies from small to large accounted for 82.4, 14.4, and 2.4%, respectively.). There was no significant difference in the relationship between polar body angle and embryo euploidy. There was no significant difference in the angular size distribution of the two polar bodies of embryos with different euploidy (*p* > 0.05).

### The relationship between the state of polar bodies and embryo euploidy

**FIGURE 3:**
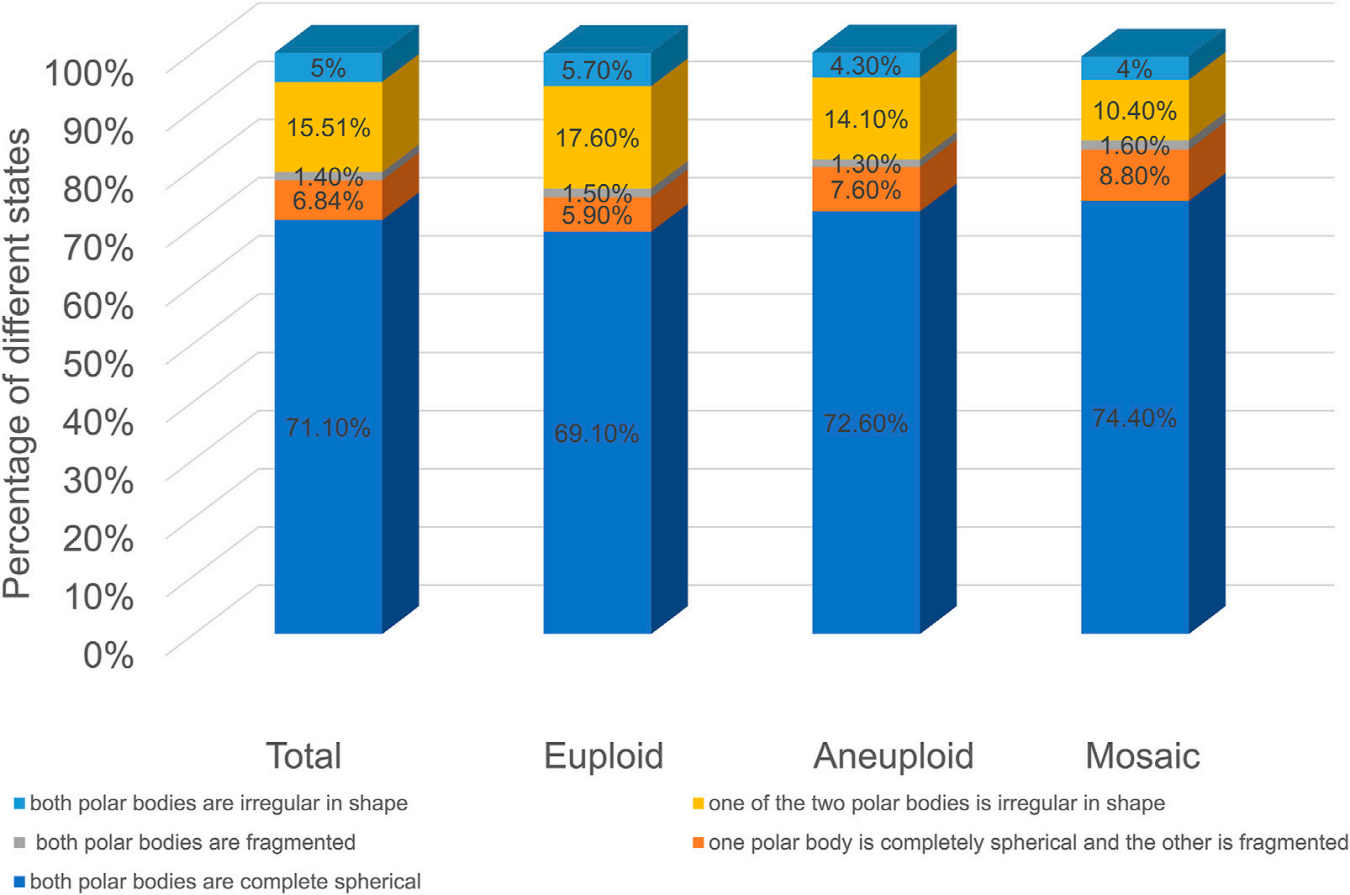
Polar body morphology and embryo euploidy.

As shown in Figure 3, among the five groups, embryos with spherical polar bodies accounted for the largest proportion, and embryos with fragmented polar bodies accounted for the least proportion (In the euploid group, the proportion of both polar bodies are complete spherical was 69.1%; the proportion of one polar body is completely spherical and the other is fragmented was 5.9%; the proportion of both polar bodies are fragmented was 1.5%; the proportion of one of the two polar bodies is irregular in shape was 17.6%; the proportion of both polar bodies are irregular in shape was 5.7%. In the aneuploid group, the proportion of both polar bodies are complete spherical was 72.6%; the proportion of one polar body is completely spherical and the other is fragmented was 7.6%; the proportion of both polar bodies are fragmented was 1.3%; the proportion of one of the two polar bodies is irregular in shape was 14.1%; the proportion of both polar bodies are irregular in shape was 4.3%. In the mosaic group, the proportion of both polar bodies are complete spherical was 74.4%; the proportion of one polar body is completely spherical and the other is fragmented was 8.8%; the proportion of both polar bodies are fragmented was 1.6%; the proportion of one of the two polar bodies is irregular in shape was 10.4%; the proportion of both polar bodies are irregular in shape was 4%). The proportions of each type of polar body in euploid, aneuploid, and chimeric embryos were the same, and there was no significant difference (*p* > 0.05).

### Time of the second polar body emission

**FIGURE 4:**
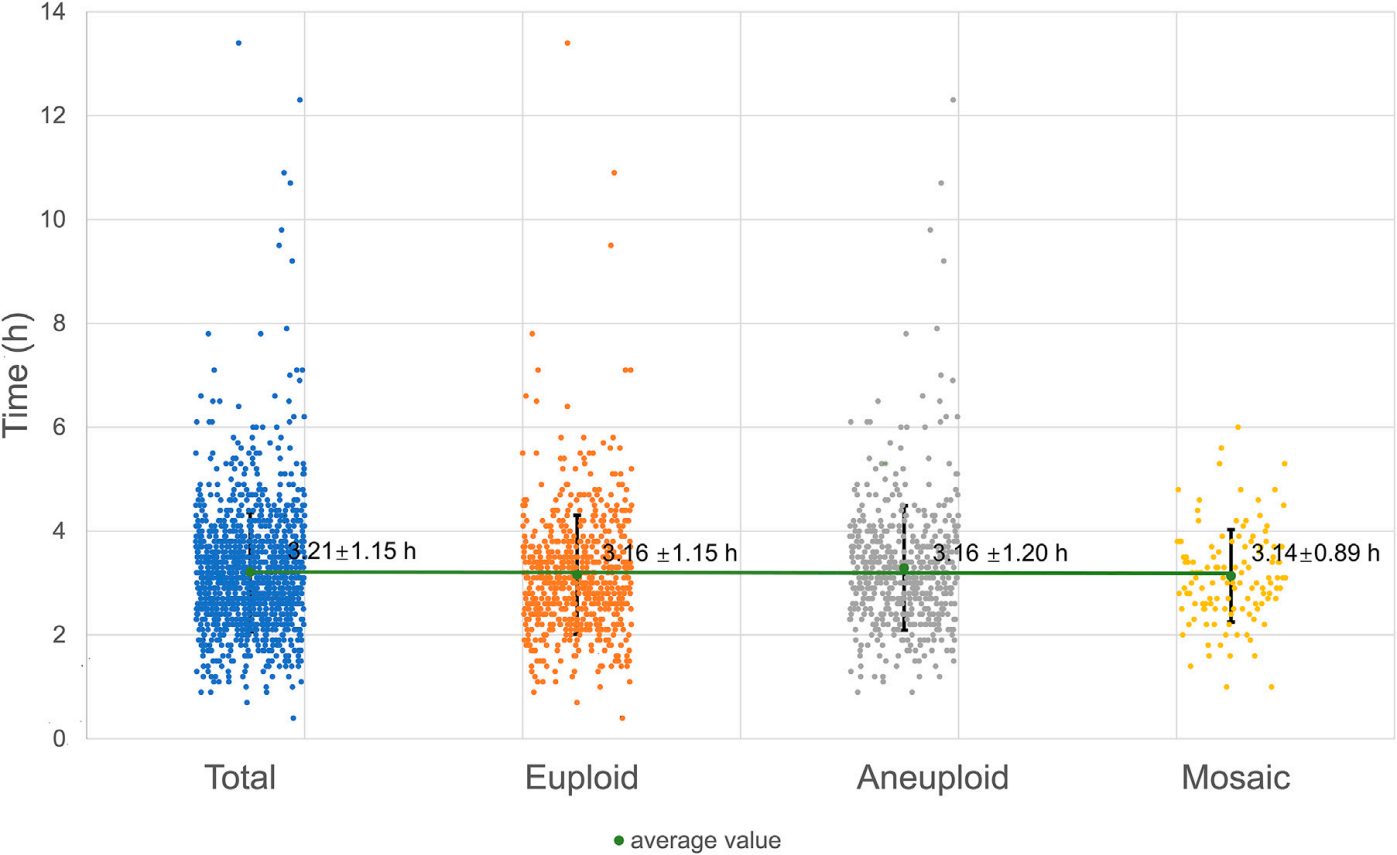
Time of the second polar body emission.

The process of second polar body exclusion can be clearly observed in embryos fertilized by ICSI cultured in the TL system. The time range distribution of the second polar body in embryos with different karyotypes was basically the same, and there was no significant difference (as shown in Figure 4).

## Discussion

In amphibian and bird species, polar body firing is a biological necessity: external embryogenesis requires the preservation of a yolk-rich cytoplasm until the feeding stage. However, as mammalian embryos implant and develop in the mother’s womb, it is unclear why polar bodies need to be created. It has been shown that removing up to half of a mouse oocyte’s cytoplasm does not affect the oocyte’s ability to fertilize and then develop to term (Wakayama et al., 1998). The most obvious reason why nature preserves polar body emissions in mammals is to preserve the unique structural integrity of functional oocytes. Fully grown oocytes are enclosed in a protein membrane, the zona pellucida (equivalent to the vitelline membrane in amphibian species). The zona pellucida provides mechanical strength to the large oocyte, ensures fertilization of a single endosperm, and protects the embryo until hatching at implantation (Paul et al., 2008). Separation of primary oocytes into two mature oocytes can lead to premature disruption of the zona pellucida, loss of oocyte integrity, or the risk of cleavage bodies mixing two individual embryos within the same zona pellucida (Otsuki et al., 2012).

We know that the same chromosomes with genetic and reproductive potential exist in the polar body as the oocyte cell. Going through two meiotic divisions, the oocyte cells expel the first and second polar bodies, respectively. The appearance of the first polar body marks the maturation of the oocyte nucleus, and the appearance of the second polar body marks the fertilization process is over of the oocyte. The first polar body can reflect the quality of oocyte cells and is related to oocyte cell fertilization, embryo quality, and post-implantation rate. This view has been widely accepted and used in practice. Theoretically, the second polar body is excreted from the fertilized oocyte cell and should be able to more directly reflect the quality of the fertilized oocyte and its subsequent embryonic development. However, studies on second polar bodies are rarely reported. Recent studies have shown that the second PB (PB2) in the mouse zygote may play roles in cell fate specification and postimplantation development. A subset of mRNAs encoding pluripotency-related factors is enriched in PB2. Nascent proteins may be synthesized in PB2 after fertilization and transported from PB2 to the zygote before the 2-cell stage. The PB2-attached blastomere (pbB) at the 2-cell stage, compared to the other blastomere (npbB), likely contributes more descendants to the inner cell mass (ICM) lineage in the blastocyst (Jin et al., 2022).

In the *in vitro* fertilization and embryo transfer technology, the selection of embryos with high developmental potential for transfer is essential to improve the pregnancy rate and reduce multiple pregnancies. At present, the evaluation of early embryonic developmental potential is mainly based on the morphological characteristics of embryos at the division stage to grade or score embryos. The developmental potential of the oocyte/embryo can be predicted based on the characteristics of the oocyte, the first polar body, and the pronucleus. Ebner et al. (Ebner et al., 2005; Rienzi et al., 2008) believed that the Shape of oocyte, cytoplasmic color, zona pellucida thickness, periocular space size, and first polar body were used to evaluate oocyte quality; However, some studies suggest that the morphological characteristics of oocytes do not fully reflect the fertilization rate and embryo quality (Albertini et al., 2003). Verlinsky (Verlinsky et al., 2003) suggested that embryonic development can be evaluated according to the different grading of the first polar body. However, the results of many studies suggest that the developmental potential of an embryo cannot be assessed based on the morphology of the first polar body (Ubaldi et al., 2008; Ebner et al., 2000; Ebner, 2002 #121; Ciotti et al., 2004).

Currently, there are few reports on the value of the second polar body in predicting embryo quality. In this paper, with the help of the TL system, the whole process of expelling the pole body, especially the expulsion process of the second pole body, can be clearly observed (Ma et al., 2022; Huang et al., 2021). We took the two polar bodies together and focused on the characteristics of polar bodies, the relationship between polar body states, and embryonic euploidy. As shown in Figure 2, as the angle between the two polar bodies increases, the proportion of embryos decreases. The proportion of embryos with an included angle less than 30° exceeds 80%, and the proportion of embryos with an included angle greater than 90° is the least, less than 3%. Euploid, aneuploid, and chimeric embryos all fit this distribution. This preliminary result shows that the position of the polar body does not seem to be directly related to its euploidy. Some studies have shown that the morphology of polar bodies is related to the quality of embryos (Lasiene et al., 2009; Navarro et al., 2009; Ubaldi et al., 2008). We divided the polar bodies into five groups according to the shape and homogeneity of the two polar bodies (as shown in Figure 3). Among them, embryos with both polar bodies were spherical in the largest proportion, and embryos with both polar bodies were fragmented in the smallest proportion. Our data suggest that embryo euploidy may not be correlated with the positional relationship of polar bodies and their morphology. This has a lot to do with the embryos we included in the study. Due to the peculiarities of clinical work, we could not include all oocytes obtained in the study. Instead, those embryos met the criteria (≥3BC) were selected as study subjects. In the selection of materials, we excluded embryos with poor quality or poor developmental potential, and this regret may have a decisive impact on our research results.

In conclusion, our results suggest that blastocyst euploidy does not correlate effectively with polar body status. Therefore, in the process of clinical embryo selection, embryologists should not predict the euploidy of blastocysts based on the state of polar bodies, thus affecting embryo selection.

## Data Availability

The original contributions presented in the study are included in the article/Supplementary Material further inquiries can be directed to the corresponding authors.

## References

[B1] Albertini D. F. Sanfins A. Combelles C. M. (2003). Origins and manifestations of oocyte maturation competencies. Reprod. Biomed. Online 6 (4), 410–415. 10.1016/s1472-6483(10)62159-1 12831584

[B2] Baltus A. E. Menke D. B. Hu Y. C. Goodheart M. L. Carpenter A. E. de Rooij D. G. (2006). In germ cells of mouse embryonic ovaries, the decision to enter meiosis precedes premeiotic DNA replication. Nat. Genet.;38(12):1430–1434. 10.1038/ng1919 17115059

[B3] Briggs D. A. Power N. J. Lamb V. Rutherford A. J. Gosden R. G. (2000). Amplification of DNA sequences in polar bodies from human oocytes for diagnosis of mitochondrial disease. Lancet 355 (9214), 1520–1521. 10.1016/s0140-6736(00)02171-1 10801178

[B4] Chen C. H. Lee C. I. Huang C. C. Chen H. H. Ho S. T. Cheng E. H. (2021). Blastocyst morphology based on uniform time-point Assessments is correlated with mosaic levels in embryos. Front. Genet. 12, 783826. 10.3389/fgene.2021.783826 35003219 PMC8727871

[B5] Chuang T. H. Chang Y. P. Lee M. J. Wang H. L. Lai H. H. Chen S. U. (2020). The incidence of mosaicism for individual chromosome in human blastocysts is correlated with chromosome length. Front. Genet. 11, 565348. 10.3389/fgene.2020.565348 33488666 PMC7815765

[B6] Ciotti P. M. Notarangelo L. Morselli-Labate A. M. Felletti V. Porcu E. Venturoli S. (2004). First polar body morphology before ICSI is not related to embryo quality or pregnancy rate. Hum. Reprod. 19 (10), 2334–2339. 10.1093/humrep/deh433 15347596

[B7] Durban M. Benet J. Boada M. Fernández E. Calafell J. M. Lailla J. M. (2001). PGD in female carriers of balanced Robertsonian and reciprocal translocations by first polar body analysis. Hum. Reprod. Update 7 (6), 591–602. 10.1093/humupd/7.6.591 11727868

[B8] Ebner T. Moser M. Sommergruber M. Gaiswinkler U. Shebl O. Jesacher K. (2005). Occurrence and developmental consequences of vacuoles throughout preimplantation development. Fertil. Steril. 83 (6), 1635–1640. 10.1016/j.fertnstert.2005.02.009 15950630

[B9] Ebner T. Yaman C. Moser M. Sommergruber M. Feichtinger O. Tews G. (2000). Prognostic value of first polar body morphology on fertilization rate and embryo quality in intracytoplasmic sperm injection. Hum. Reprod. 15 (2), 427–430. 10.1093/humrep/15.2.427 10655316

[B10] Gardner D. K. Schoolcraft W. B. (1999). Culture and transfer of human blastocysts. Curr. Opin. Obstet. Gynecol. 11 (3), 307–311. 10.1097/00001703-199906000-00013 10369209

[B11] Gutiérrez-Mateo C. Benet J. Wells D. Colls P. Bermúdez M. G. Sánchez-García J. F. (2004). Aneuploidy study of human oocytes first polar body comparative genomic hybridization and metaphase II fluorescence *in situ* hybridization analysis. Hum. Reprod. 19 (12), 2859–2868. 10.1093/humrep/deh515 15520023

[B12] Huang B. Hu D. Qian K. Ai J. Li Y. Jin L. (2014). Is frozen embryo transfer cycle associated with a significantly lower incidence of ectopic pregnancy? An analysis of more than 30, 000 cycles. Fertil. Steril. 102 (5), 1345–1349. 10.1016/j.fertnstert.2014.07.1245 25241365

[B13] Huang B. Qian K. Li Z. Yue J. Yang W. Zhu G. (2015). Neonatal outcomes after early rescue intracytoplasmic sperm injection: An analysis of a 5-year period. Fertil. Steril. 103 (6), 1432–1437. 10.1016/j.fertnstert.2015.02.026 25813286

[B14] Huang B. Tan W. Li Z. Jin L. (2021). An artificial intelligence model (euploid prediction algorithm) can predict embryo ploidy status based on time-lapse data. Reprod. Biol. Endocrinol. 19 (1), 185. 10.1186/s12958-021-00864-4 34903224 PMC8667440

[B15] Jin H. Han Y. Wang H. Li J. Shen W. Zhang L. (2022). The second polar body contributes to the fate asymmetry in the mouse embryo. Natl. Sci. Rev. 9, nwac003. 10.1093/nsr/nwac003 35919785 PMC9337984

[B16] Knoblich J. A. (2001). Asymmetric cell division during animal development. Nat. Rev. Mol. Cell Biol. 2 (1), 11–20. 10.1038/35048085 11413461

[B17] Kudo N. R. Wassmann K. Anger M. Schuh M. Wirth K. G. Xu H. (2006). Resolution of chiasmata in oocytes requires separase-mediated proteolysis. Cell 126 (1), 135–146. 10.1016/j.cell.2006.05.033 16839882

[B18] Lasiene K. Vitkus A. Valanciūte A. Lasys V. (2009). Morphological criteria of oocyte quality. Med. Kaunas. 45 (7), 509–515. 10.3390/medicina45070067 19667744

[B19] Lee J. Okada K. Ogushi S. Miyano T. Miyake M. Yamashita M. (2006). Loss of Rec8 from chromosome arm and centromere region is required for homologous chromosome separation and sister chromatid separation, respectively, in mammalian meiosis. Cell Cycle 5 (13), 1448–1455. 10.4161/cc.5.13.2903 16855401

[B20] Liu X. J. (2012). Polar body emission. Cytoskeleton 69 (10), 670–685. 10.1002/cm.21041 22730245

[B21] Ma B. X. Zhang H. Jin L. Huang B. (2022). Neonatal outcomes of embryos cultured in a time-lapse incubation system: An analysis of more than 15, 000 fresh transfer cycles. Reprod. Sci. 29 (5), 1524–1530. 10.1007/s43032-021-00714-z 34406638

[B22] Navarro P. A. de Araújo M. M. de Araújo C. M. Rocha M. dos Reis R. Martins W. (2009). Relationship between first polar body morphology before intracytoplasmic sperm injection and fertilization rate, cleavage rate, and embryo quality. Int. J. Gynaecol. Obstet. 104 (3), 226–229. 10.1016/j.ijgo.2008.11.008 19105998

[B23] Otsuki J. Nagai Y. Lopata A. Chiba K. Yasmin L. Sankai T. (2012). Symmetrical division of mouse oocytes during meiotic maturation can lead to the development of twin embryos that amalgamate to form a chimeric hermaphrodite. Hum. Reprod. 27 (2), 380–387. 10.1093/humrep/der408 22147919

[B24] Paul W. (2008). Zona pellucida glycoproteins. J. Biol. Chem. 283 (36), 24285–24289. 10.1074/jbc.R800027200 18539589 PMC2528931

[B25] Revenkova E. Herrmann K. Adelfalk C. Jessberger R. (2010). Oocyte cohesin expression restricted to predictyate stages provides full fertility and prevents aneuploidy. Curr. Biol. 20 (17), 1529–1533. 10.1016/j.cub.2010.08.024 20817531 PMC2945217

[B26] Rienzi L. Ubaldi F. M. Iacobelli M. Minasi M. G. Romano S. Ferrero S. (2008). Significance of metaphase II human oocyte morphology on ICSI outcome. Fertil. Steril. 90 (5), 1692–1700. 10.1016/j.fertnstert.2007.09.024 18249393

[B27] Ubaldi F. Rienzi L. (2008). Morphological selection of gametes. Placenta 29 (Suppl. B), 115–120. 10.1016/j.placenta.2008.08.009 18762336

[B28] Uhlmann F. Nasmyth K. (1998). Cohesion between sister chromatids must be established during DNA replication. Curr. Biol. 8 (20), 1095–1101. 10.1016/s0960-9822(98)70463-4 9778527

[B29] Verlinsky Y. Lerner S. Illkevitch N. Kuznetsov V. Kuznetsov I. Cieslak J. (2003). Is there any predictive value of first polar body morphology for embryo genotype or developmental potential? Reprod. Biomed. Online 7 (3), 336–341. 10.1016/s1472-6483(10)61874-3 14653896

[B30] Verlinsky Y. Rechitsky S. Cieslak J. Ivakhnenko V. Wolf G. Lifchez A. (1997). Preimplantation diagnosis of single gene disorders by two-step oocyte genetic analysis using first and second polar body. Biochem. Mol. Med. 62 (2), 182–187. 10.1006/bmme.1997.2635 9441871

[B31] Wakayama T. Yanagimachi R. (1998). Fertilisability and developmental ability of mouse oocytes with reduced amounts of cytoplasm. Zygote 6 (4), 341–346. 10.1017/s096719949800029x 9921644

[B32] Wu L. Jin L. Chen W. Liu J. M. Hu J. Yu Q. (2021). The true incidence of chromosomal mosaicism after preimplantation genetic testing is much lower than that indicated by trophectoderm biopsy. Hum. Reprod. 36 (6), 1691–1701. 10.1093/humrep/deab064 33860322

[B33] Yang D. Feng D. Gao Y. Sagnelli M. Wang X. Li D. (2020). An effective method for trophectoderm biopsy using mechanical blunt dissection: A step-by-step demonstration. Fertil. Steril. 114 (2), 438–439. 10.1016/j.fertnstert.2020.05.035 32654814

[B34] Zong C. Lu S. Chapman A. R. Xie X. S. (2012). Genome-wide detection of single-nucleotide and copy-number variations of a single human cell. Science 338 (6114), 1622–1626. 10.1126/science.1229164 23258894 PMC3600412

